# Dose-dependent expression of neuronal injury markers during experimental osteoarthritis induced by monoiodoacetate in the rat

**DOI:** 10.1186/1744-8069-8-50

**Published:** 2012-07-08

**Authors:** Joana Ferreira-Gomes, Sara Adães, Raquel Meireles Sousa, Marcelo Mendonça, José Manuel Castro-Lopes

**Affiliations:** 1 Department of Experimental Biology, Faculty of Medicine of Porto and IBMC - Institute for Molecular and Cell Biology, University of Porto, Alameda Prof. Hernani Monteiro, Porto, 4200-319, Portugal

**Keywords:** Osteoarthritis, Pain, Mono-iodoacetate, ATF-3, NPY, GAP-43, Neuronal injury

## Abstract

**Background:**

It was recently reported that the mono-iodoacetate (MIA) experimental model of osteoarthritis (OA) courses with changes of neurons innervating the affected joints that are commonly interpreted as a neuronal response to axonal injury. To better characterize these changes, we evaluated the expression of two markers of neuronal damage, ATF-3 and NPY, and the growth associated protein GAP-43, in primary afferent neurons of OA animals injected with three different doses of MIA (0.3, 1 or 2 mg). Measurements were performed at days 3, 7, 14, 21 and 31 post-MIA injection.

**Results:**

OA animals showed the characteristic histopathological changes of the joints and the accompanying nociceptive behaviour, evaluated by the Knee-Bed and CatWalk tests. An increase of ATF-3 expression was detected in the DRG of OA animals as early as 3 days after the injection of 1 or 2 mg of MIA and 7 days after the injection of 0.3 mg. NPY expression was increased in animals injected with 1 or 2 mg of MIA, at day 3 or in all time-points, respectively. From day 7 onwards there was a massive increase of GAP-43 expression in ATF-3 cells.

**Conclusions:**

The expression of the neuronal injury markers ATF-3 and NPY as well as an up-regulation of GAP-43 expression, indicative of peripheral fibre regeneration, suggests that axonal injury and a regeneration response may be happening in this model of OA. This opens new perspectives in the unravelling of the physiopathology of the human disease.

## Background

Osteoarthritis (OA) is a chronic degenerative joint disorder that affects a large proportion of the population, being the most common type of articular disorders. Patients’ major clinical manifestation is chronic pain that typically worsens with weight bearing and activity or movement of the affected joint [1]. Joint pain results from the activation of primary afferent nerve fibres at the joint, but the exact mechanisms of pain in OA remain inadequately understood [2,3].

Several studies in animal models of OA have been performed to unravel the nociceptive mechanisms in this pathology. The experimental model most commonly used is the intra-articular injection of the metabolic inhibitor mono-iodoacetate (MIA) into the knee joint of the rat. This compound inhibits the activity of glyceraldehyde-3-phosphate dehydrogenase of articular chondrocytes, leading to disruption of glycolytic energy metabolism and synthetic processes and eventually to cell death [4,5]. Hence, a progressive loss of articular cartilage and lesions of the subchondral bone are observed in this model, which have been described as closely resembling those observed in OA patients [4].

A marker of neuronal damage, ATF-3, has been shown in dorsal root ganglia (DRG) neurons, 8 and 14 days post-MIA injection [6]. Moreover, we observed an increase in CGRP in DRG cells 31 days after MIA injection, which occurred mainly in the medium and large cell size population that in control animals did not express this peptide [7]. This phenomenon could result from a phenotypic switch of these cells to assume characteristics of nociceptors, or from a hypertrophy of small or medium size cells that already expressed CGRP, since an alteration of the total cell size distribution pattern was also observed [7]. Notwithstanding, both phenomena have been described as a neuronal response to axonal injury [8-11]. Furthermore, we also observed a reduced retrograde labelling of Fluorogold in DRG neurons of OA animals following injection of the tracer into the affected knee, which could be due to axonal loss or retraction, or another form of injury of the nerve terminals [7]. These data raise the question of a possible damage of the joint afferents that would provoke changes in the soma of these neurons, at the DRG level, that resemble those occurring during a peripheral neuropathy.

To better understand and characterize the possible occurrence of neuronal injury in the MIA model of OA, we evaluated the expression of ATF-3 and NPY in DRG neurons at different time-points of disease progression, from 3 days after MIA injection until 31 days. ATF-3 is a transcription factor that, as previously mentioned, has been considered a marker of neuronal damage since it is elevated in the DRG neurons following peripheral axonal injury [12,13]. Similarly, NPY is a neurotransmitter that under normal conditions is barely detected in DRG neurons [14,15], but peripheral nerve injury induces its expression in their cell bodies [16,17]. Additionally, we investigated whether a regeneration process was occurring as part of a neuronal response to a hypothetical injury of peripheral nerves. The growth-associated protein GAP-43 served that purpose since its expression is up-regulated in regenerating peripheral fibres [18-20]. Finally, since there is a correlation between MIA concentration and the degree of sensitization of afferent nerve fibres [21], three different doses of MIA were used in this study in order to investigate whether the observed effects were dose-dependent.

## Results

### Histological analysis of the knee joint

**Figure 1:**
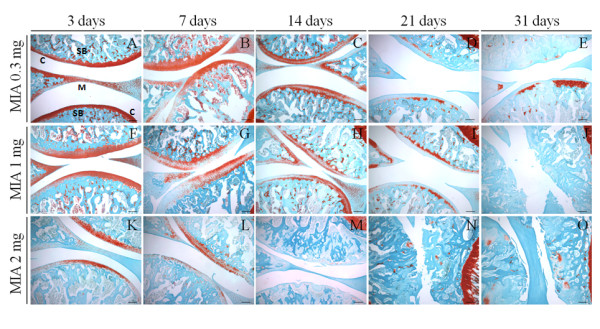
**Histopathology of knee sections of OA animals.** Rats received an intra-articular injection of 0.3 (**A**-**E**), 1 (**F**-**J**) or 2 mg (**K**-**O**) of MIA and were sacrificed at 3 (A, F, K), 7 (B, G, L), 14 (C, H, M), 21 (D, I, N) and 31 days (E, J, O) post-MIA injection. Each rat knee image is representative of a group of 5 animals. The sections were stained with Safranin-O that stained cartilage and its proteoglycan content and Fast Green, which stains bone. Histopathological alterations, namely decrease in proteoglycan staining, chondrocyte death, loss of intercellular matrix, decrease of the thickness of the articular cartilage, erosion of the hyaline articular cartilage and exposure of the subcondral bone, were dose and time-dependent. C indicates cartilage; SB, subchondral bone; M, meniscus. Scale bar: 200 μm.

No damage of the knee joint could be observed in saline-injected control animals at all time-points studied (data not shown). In contrast, the histopathological findings observed in OA animals were dose and time-dependent. At day 3, no alteration or a minimal decrease in the proteoglycan staining was observed in animals injected with 0.3 mg of MIA, resulting in a slight decrease of Safranin-O staining (Figure 1A). This became more apparent at day 14, when some irregularity in the superficial zone of the articular cartilage was observed (Figure 1C). At day 21, chondrocyte death and loss of intercellular matrix was observed (Figure 1D), and this was more pronounced and accompanied by a marked decrease of the thickness of the articular cartilage at day 31 (Figure 1E).

A minimal decrease in the proteoglycan staining was observed at day 3 in animals injected with 1 mg of MIA, becoming more pronounced at day 7 (Figure 1F-G). At day 14, some chondrocyte death and loss of matrix led to decreased thickness of the articular cartilage, that was more evident at day 21 (Figure 1H-I). At day 31, the articular surface showed fissures and subchondral bone become exposed (Figure 1J).

With the higher dose of MIA (2 mg), proteoglycan staining loss from the extracellular matrix of the articular cartilage was more pronounced at day 3 than with the lower doses (Figure 1K). Moreover, chondrocyte death was already apparent at day 7, and at day 14 there was a marked loss of extracellular matrix and a decrease of the thickness of the articular cartilage, which presented some fissures (Figure 1L-M). A complete erosion of the hyaline articular cartilage and exposure of the subcondral bone was observed at day 21, while thickening of the subchondral bone was apparent at day 31 (Figure 1N-O).

### Behavioural testing

**Figure 2:**
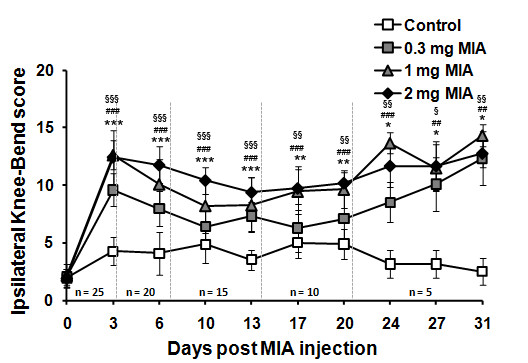
**Evolution of the Knee-Bend score of animals injected with saline (control; n = 5) or 0.3, 1 or 2 mg of MIA (OA; n = 25/group) on various days after the injection until the day of sacrifice (3, 7, 14, 21, 31).** At each sacrifice time-point 5 animals per group were euthanized. Baseline score was determined for all animals prior to the injection (day 0). * P < 0.05, ** P < 0.01, *** P < 0.001 significantly different from baseline values for the 0.3 mg dose of MIA; ## P < 0.01, ### P < 0.001 significantly different from baseline values for the 1 mg dose of MIA; § P < 0.05, §§ P < 0.01, §§§ P < 0.001 significantly different from baseline values for the 2 mg dose of MIA (Repeated Measures ANOVA followed by LSD post-hoc test).

**Figure 3:**
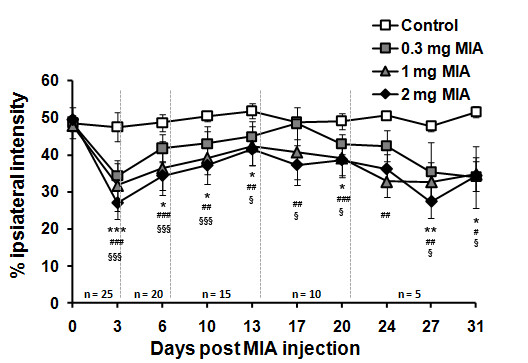
**Evolution of the percentage of the ipsilateral paw-print total intensity, assessed in the CatWalk test, of animals injected with saline (control; n = 5) or 0.3, 1 or 2 mg of MIA (OA; n = 25/group) on various days after the injection until the day of sacrifice (3, 7, 14, 21, 31)**. At each sacrifice time-point, 5 animals per group were euthanized. Baseline score was determined for all animals prior to injection (day 0). * P < 0.05, *** P < 0.001 significantly different from baseline values for the 0.3 mg dose of MIA; # P < 0.05, ## P < 0.01, ### P < 0.001 significantly different from baseline values for the 1 mg dose of MIA; § P < 0.05, §§§ P < 0.001 significantly different from baseline values for the 2 mg dose of MIA (Repeated Measures ANOVA followed by LSD post-hoc test).

Knee-Bend and CatWalk tests were used to evaluate the movement-induced nociception caused by different doses of MIA. Overall, changes in nociception were concentration- and time-dependent (Figures 2 and 3).

Knee-Bend scores of saline-injected control animals were similar to those observed at baseline in all time-points studied. When compared to the baseline values and to the control animals, an increase in the Knee-Bend score was evident as early as day 3 post-injection for the 3 concentrations of MIA (Figure 2). The animals receiving 0.3 mg of MIA showed the lowest Knee-Bend score. The score, which was significantly higher than baseline levels at all time-points studied (P<0.05; Figure 2), was increased at day 3, slightly reducing from then until day 17, when it started to increase again.

Animals receiving 1 or 2 mg of MIA showed a similar high Knee-Bend score at day 3 (Figure 2). After this initial phase of disease progression, Knee-Bend scores of animals injected with 2 mg were higher than those of animals injected with 1 mg, while at latter stages no differences were observed. In fact, the Knee-Bend scores of animals injected with 1 mg of MIA showed an evolution pattern similar to that observed with the 0.3 mg concentration (Figure 2), with a marked increase at day 3, reducing from that time-point till day 13, and a further increase at day 17, reaching values similar to day 3 from day 24 onwards. The evolution pattern of the animals injected with 2 mg of MIA was smoother, reducing very slightly after day 3 and returning to these levels from day 24 onwards (Figure 2). At day 31, animals receiving the three different doses showed similar Knee-Bend scores.

The percentage of ipsilateral paw-print intensity, as measured by the CatWalk test, followed a pattern that was also concentration- and time-dependent (Figure 3). Animals injected with 0.3 mg of MIA showed the least decrease in the ipsilateral paw-print intensity. At most time-points, the ipsilateral paw-print intensity of these animals were significantly below baseline levels and below levels of control animals, being not significantly different from baseline values only at days 17 and 24 (Figure 3). Animals injected with 1 or 2 mg of MIA showed a reduced ipsilateral paw-print intensity significantly different from baseline levels and from control animals at all time-points evaluated (P<0.05; Figure 3), which was more pronounced in the animals injected with 2 mg (with the exception of day 24 that was not significantly different for this dose).

The temporal profile observed in the CatWalk test was similar for the three MIA doses. There was an initial decrease in the ipsilateral paw-print intensity at day 3, followed by a slight increase until days 13–17, decreasing thereafter. At day 31, the ipsilateral paw-print intensity observed was similar for the OA animals injected with the different MIA doses (Figure 3).

No differences in the ipsilateral paw-print intensity were observed in the saline-injected animals throughout the study.

### Immunohistochemistry

**Figure 4:**
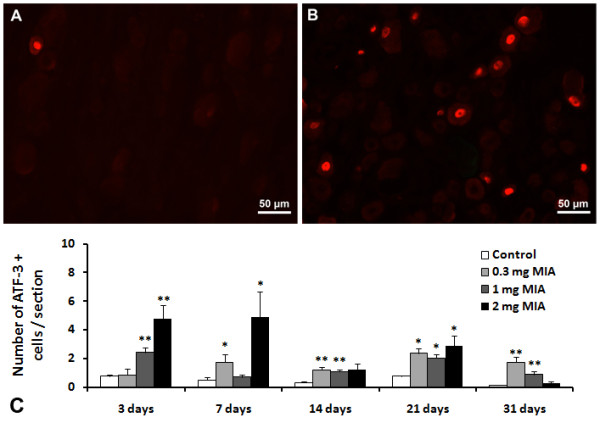
**ATF-3 expression in L3, L4 and L5 ipsilateral DRG.** Animals received an intra-articular injection of saline (control) or 0.3, 1 or 2 mg of MIA (OA) and were sacrificed at 3, 7, 14, 21 and 31 days post-injection. A and B are images representative of immunofluorescence reactions for ATF-3 from control rats (**A**) and rats injected with 2 mg of MIA (**B**) sacrificed at 7 days. * P < 0.05, ** P < 0.01 significantly different from controls (Mann–Whitney test).

**Figure 5:**
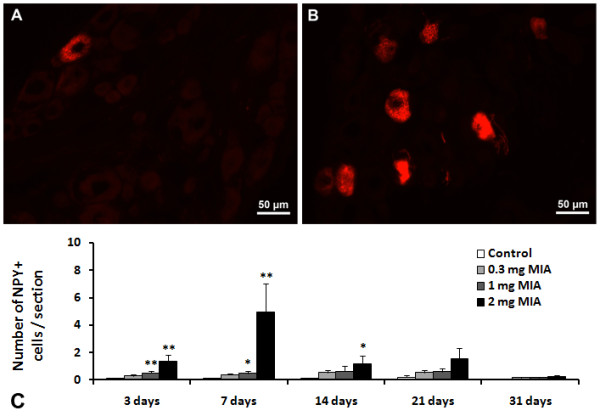
**NPY expression in L3, L4 and L5 ipsilateral DRG.** Animals received an intra-articular injection of saline (control) or 0.3, 1 or 2 mg of MIA (OA) and were sacrificed at 3, 7, 14, 21 and 31 days post-injection. A and B are images representative of immunofluorescence reactions for NPY from control rats (**A**) and rats injected with 2 mg of MIA (**B**) sacrificed at 7 days. * P < 0.05, ** P < 0.01 significantly different from controls (Mann–Whitney test).

The number of neurons expressing ATF-3 and NPY was analysed in animals injected with saline and with 0.3, 1 or 2 mg of MIA, at days 3, 7, 14, 21 and 31 post-injection. FG was injected in all animals 7 days prior to sacrifice. Because both ATF-3 and NPY expression was observed in both FG positive and FG negative cells, and since, as already mentioned, a reduced retrograde labelling of FG in the DRG of OA animals was observed [7], we chose to show the numerical data as the total number of neurons expressing ATF-3 or NPY per section rather than the percentage of FG labelled cells that were immunopositive. The temporal profile and dose-related expression was different for the two markers evaluated (Figures 4C and 5C).

**Figure 6:**
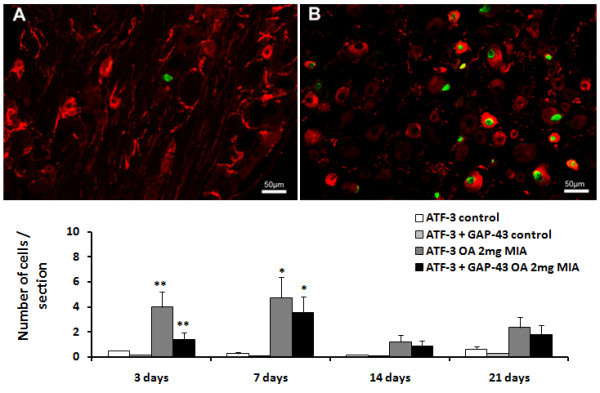
**GAP-43 expression in ATF-3 positive cells in L3, L4 and L5 ipsilateral DRG.** Animals received an intra-articular injection of saline (control) or 2 mg of MIA (OA) and were sacrificed at 3, 7, 14 and 21 days post-injection. A and B are images representative of immunofluorescence reactions for ATF-3 plus GAP-43 in sections of ipsilateral DRG from control rats (**A**) and rats injected with 2 mg of MIA (**B**) sacrificed at 7 days.

The expression of GAP-43 was evaluated in ATF-3 positive cells, in animals injected with saline and with 2 mg of MIA, at days 3, 7, 14 and 21 post-injection (Figures 6). Since at 31 days ATF-3 expression in the 2 mg group of animals is at baseline levels, this time-point was excluded in this analysis.

### Expression of ATF-3 in DRGs during MIA-induced OA

Baseline ATF-3 expression was similar in control animals at all time-points studied in both the ipsilateral and contralateral DRG, as well as in the contralateral DRG of OA animals. There was an increase of the number of neurons expressing ATF-3 in ipsilateral DRG of MIA-injected animals, which was dose and time-dependent (Figure 4). At day 3, no changes were observed in animals injected with 0.3 mg of MIA, but at day 7 there was an upregulation of the number of neurons expressing ATF-3 in the DRG (P<0.05; Figure 4). This upregulation was maintained at day 14, increasing further at days 21 and 31.

Animals injected with 1 mg of MIA showed an increase of the number of neurons expressing ATF-3 significantly different from control animals as early as day 3 (from 0.8 ± 0.1 cells/section in control animals to 2.4 ± 0.4 cells/section in OA animals; P<0.01). The number of neurons expressing ATF-3 declined slightly until day 14, increasing again at day 21. At day 31, the number of neurons expressing ATF-3 decreased again, though it was significantly higher than the baseline expression observed in control animals (P<0.01; Figure 4).

The dose of 2 mg of MIA induced a marked increase in the number of neurons expressing ATF-3 at day 3 (from 0.8 ± 0.1 cells/section in control animals to 4.8 ± 0.9 cells/section in OA animals; P<0.01). This upregulation was maintained at day 7, decreasing significantly to baseline levels at day 14. At day 21, a further increase of the number of neurons expressing ATF-3 was observed (2.9 ± 0.7 ATF-3 positive cells/section), significantly different from control animals (P<0.05; Figure 4). At day 31, the number of neurons expressing ATF-3 returned again to baseline levels.

**Figure 7:**
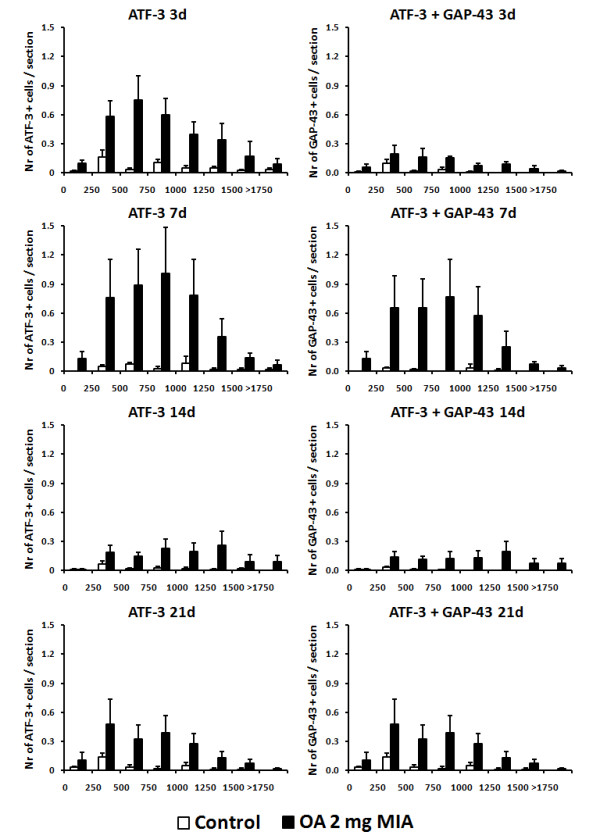
**ATF-3 and GAP-43 plus ATF-3 expression per neuronal perikaria cross-sectional area range of control and 2 mg of MIA OA animals.** The DRGs were evaluated at 3, 7, 14 and 21 days post-injection.

The number of neurons expressing ATF-3 was higher with the 2 mg dose of MIA, therefore evaluation of cell size range was performed in these animals. ATF-3 expression was observed across all neuronal size ranges (Figure 7). At 3 and 7 days the expression followed the normal distribution pattern, but at day 14, when there was a decrease of the number of neurons expressing ATF-3, it was as a consequence of decreased expression in small and medium size cells (Figure 7).

### Expression of NPY in DRGs during MIA-induced OA

Similarly to ATF-3 expression, the increase of NPY expression profile was dose and time-dependent (Figure 5). A very low baseline expression was observed in control animals at all time-points studied, and in the contralateral DRG of OA animals (data not shown). In the animals injected with 0.3 mg of MIA, the number of neurons expressing NPY in the ipsilateral DRG was at baseline levels at day 3. Thereafter, a slight increase of the number of neurons expressing NPY occurred until days 14 and 21, decreasing again at day 31. Nevertheless, none of these very modest differences reached statistical significance (Figure 5).

Conversely, an increase in the number of neurons expressing NPY was observed at day 3 in the animals injected with 1 mg of MIA (from 0.1 ± 0.0 cells/section in control animals to 0.5 ± 0.1 cells/section in OA animals; P<0.01). The number of neurons expressing NPY maintained thereafter, returning to baseline levels at 31 days (Figure 5).

The dose of 2 mg of MIA induced a significant increase of the number of neurons expressing NPY immediately at day 3, which was more striking at day 7 (with 5.0 ± 2.1 cells/section; P<0.01; Figure 5). At day 14, the number of NPY cells were similar to that observed at day 3, remaining sustained at day 21. At day 31, the number of neurons expressing NPY was similar to control animals.

As for ATF-3 expression, NPY expression was observed across all neuronal size range and at all time-points followed the normal distribution pattern (data not shown).

### Correlation between ATF-3 and NPY with behavioural data during MIA-induced OA

In an attempt to identify a possible correlation between behaviour and the markers of neuronal damage studied, a Pearson’s correlation coefficient was calculated for all time-points and doses studied. Occasional isolated parameters show significant correlation but overall no significant correlation was found between the parameters.

### Expression of GAP-43, in ATF-3 positive cells, in DRGs during MIA-induced OA

The number of neurons expressing ATF-3 was similar in single and double immunohistochemistry reactions (Figures 4 and 6). Control animals showed a very low baseline number of neurons expressing ATF-3, an average of 0.4 cells/section, and of this between 33-47% of ATF-3 positive cells were also immunoreactive for GAP-43 (0.1 ± 0.02 cells/section to 0.3 ± 0.02 cells/section) (Figure 6).

In OA animals injected with 2 mg of MIA, at 3 days post-injection of the 4.0 ± 1.2 ATF-3 cells/section, 34% were also GAP-43 positive (1.4 ± 0.6 ATF-3 + GAP43 cells/section; Figure 6), a percentage similar to that of controls. However, at day 7, of the 4.8 ± 1.6 ATF-3 cells/section, 3.6 ± 1.3 were also GAP-43 positive, which corresponds to 75% of the total ATF-3 cells. This percentage was very similar at 14 days (71%) and at 21 days (76%), despite the oscillations of the ATF-3 expression, due to the biphasic pattern observed (Figure 6).

Analysis of cell size distribution showed that ATF-3 cells that are GAP-43 positive followed the same pattern as total ATF-3 cells (Figure 7).

## Discussion

Animal models are important and essential tools to unravel the molecular mechanisms of pain in OA and for pre-clinical testing of new therapies. The MIA model has been widely used in pain associated studies and to test potential analgesic agents, since it is a rapid and easily reproducible method, and has been described as the one that best mimics the histopathology of human OA [22-26]. The severity of the MIA-induced OA has been shown to be concentration and time-dependent in a range of concentrations [22,26,27]. Upon evaluation of the histopathology profile with different doses across a time period of 31 days, we observed a pattern of lesions clearly dependent of MIA concentration. Behavioural findings also showed a dose and time-dependent pattern on nociception, when evaluated by the Knee-Bend and CatWalk tests. Moreover, the ipsilateral paw-print intensity profile here observed with the three doses of MIA was comparable to the weight bearing evaluation described by Pomonis et al. [26] using similar doses.

Neuronal hypertrophy and phenotypic alterations in animals injected with 2 mg of MIA [7], along with data revealing ATF-3 expression in DRG neurons of animals with MIA-induced OA [6], suggested that neuronal damage might be occurring in this experimental model. In the present study, we further evaluated this possibility by investigating the expression of two widely used neuronal injury markers, ATF3 and NPY, in the DRG neurons, at different time-points of the OA evolution and using different doses of MIA.

The presence of ATF-3 has been reported in L5 DRG neurons of OA animals injected with 1 mg of MIA at day 8 and 14 [6]. In another study ATF-3 expression has been reported in FG labelled L4 DRG neurons of OA animals injected with 2 mg of MIA only from day 14 onwards [28]. However, in the present study, we observed ATF-3 expression in L3, L4 and L5 DRG as early as 3 days post-MIA injection, at doses routinely used to induce OA (1 and 2 mg). Moreover, ATF-3 expression was dose and time-dependent, with a biphasic pattern, and occurred in various sized neurons as previously described in axotomized DRG neurons [13].

ATF-3 has been described by several authors as a sensitive marker of nerve damage [12,13]. This transcription factor, barely present in the DRG of naive animals, is dramatically induced in DRG neurons following peripheral axotomy [13]. Therefore, the induction of ATF-3 seems to be a cellular response to some types of stress, and nociceptive stimuli without nerve injury does not seem to induce ATF-3 in sensory neurons [12,13]. This has been corroborated in studies where sciatic nerve transection induced strong ATF-3 expression, while intra-plantar inflammation induced by Complete Freund’s Adjuvant did not [12,13]. As Braz and Basbaum [12] stress, ATF-3 expression is not triggered only by the increased activity of sensory fibres due to the lesion, nerve damage being mandatory for such expression. In MIA-induced OA there is no damage of the nerve per se, but there might be injury of nerve endings located at the joint that trigger a similar response but at a smaller scale. Similarly, intraplantar injection of formalin, causing not only tissue inflammation but also injury of local nerve endings, causes ATF-3 expression in some neurons [13]. Also, Hill et al. [29] reported that skin incision without real nerve damage induces nerve injury-like responses such as ATF-3 expression.

ATF-3 is also expressed in the DRG neurons in collagen-induced arthritis [30] and in monoarthritis following complete Freund´s adjuvant injection in the tibiotarsal joint [31]. A possible role of positive regulatory factors, such as tumour necrosis factor α (TNF-α) present in the neuroinflammatory environment, could account for the activation of ATF-3 in those conditions, as previously suggested by others [29,32]. However, it should be noted that in collagen-induced arthritis ATF-3 expression was not affected by anti-TNF therapy [30]. Furthermore, bone destruction occurs in both models [33,34] and in CFA induced arthritis there is a highly significant correlation between pain behaviour and joint destruction [34].

NPY expression in DRG neurons is evoked by injury to sensory neurons [16,17,35], therefore, expression of NPY was also evaluated in the present study. NPY has been described to contribute to the excitability of axotomized sensory neurons, which in turn could invoke aberrant spontaneous activity in damaged sensory nerves that contribute to neuropathic pain [35,36]. Furthermore, it has been suggested that NPY can be released intraganglionically, especially after peripheral nerve injury, and act as a mediator of chemical cell-to-cell signalling [15]. Conversely, painful inflammation of rats’ hind paw does not induce NPY expression in DRG neurons [37,38]. In our study, NPY expression was induced by 1 or 2 mg of MIA immediately at day 3. From day 3 forward, NPY expression was only significantly different from controls with the 2 mg dose, while the lowest dose used (0.3 mg) never induced sufficient NPY expression to reach statistical significance.

The temporal profile and dose related expression was different for the two markers evaluated in this study, which may be due to different activation mechanisms. In fact, while ATF-3 expression is a transcription factor that may be elicited immediately in DRG neurons, NPY expression might need more time since it could be dependent of sympathetic-sensory coupling within the DRG [39].

Interestingly, a biphasic pattern was observed both on behavioural data and on the expression of the injury related molecules, though no significant correlation between them was found.

Injury due to mechanical activation of the primary afferent endings present in subcondral bone has been proposed as one of the important mechanisms for the generation of pain in OA [40-42]. Considering that ATF-3 and NPY expression are signalling neuronal injury, that could explain the second wave of increased expression of ATF-3 and NPY observed in the present study at latter time points of the disease, when erosion of the cartilage and exposure of subcondral bone occurs. The absence of articular cartilage leads to bone-to-bone articulation and exposure of nociceptor endings in the bone to biomechanical forces associated with weight bearing [42]. However, both neuronal injury markers showed increased expression at day 3 with the higher doses of MIA. Day 3 corresponds to an initial phase of the disease when an inflammatory component has been described by some authors [22,23,26], and no degeneration of the cartilage is present, even with the dose of 2 mg, as observed in the histopathological analysis. A possible explanation for the increased expression of ATF-3 and NPY at this early time point could be an injury to nerve endings in the capsule and synovium. As mentioned above, some authors propose that the neuroinflammatory environment could be the trigger to this increased expression [29,31,32], but another possibility is that MIA itself may cause a chemical injury of nerve terminals in the injected knee. In fact, MIA is an inhibitor of glicolysis that ultimately leads to the necrosis of chondrocytes, but is not cell specific, and depending on the concentration used different degrees of cell death can be achieved. Actually, we observe that increasing doses of MIA induce the expression of ATF-3 and NPY in further neurons, which suggests that the chemical stimuli might induce some axonal damage producing a nerve-injury like response. In what concerns the reduction in ATF3- and NPY-expressing neurons in some time-points, the possibility of loss of knee-joint afferent neurons does not seem to explain it, since in our previous study [7] we did not observed a reduced number of neurons in DRG of MIA injected animals, when total counts were performed at 31 days.

The lack of correlation between neuropathic markers and pain behaviours previously referred, indicate that although a nerve injury may be important for pain derived from OA, other mechanisms might also contribute for nociception. In this context, the inflammatory component is likely to cause a rise in the activation of joint nociceptors, that in turn lead to an increased sensitivity of spinal cord neurons, resulting in enhanced nociception.

After peripheral axonal injury, the perikaria of affected neurons and the surrounding glial cells respond to the insult with morphological, metabolic and biochemical changes [43], in order to promote survival and regeneration of the lesioned nerves [44]. ATF-3, besides being implicated in cell death, may also have a role in inhibition of apoptosis and induction of neurite elongation, and thus promote neuronal survival, depending on the stress signal, cell type and intracellular pathway activated [45]. In fact, in the oncology field, where ATF-3 has been extensively studied, it seems that the cellular context strongly influences its role in cancer development, acting as an oncogene or as a tumour suppressor [46]. Actually, it is possible that the fluctuation of ATF-3 over time has to do with the trigger signal, and also derives from the fact that ATF-3 expression is transient and regulates the balance between proliferative and apoptotic signals.

On the other hand, GAP-43 expression seems to peak when axons are elongating [47]. Therefore, GAP-43 expression was analysed in order to evaluate whether an enhanced growth state had been activated. An increased expression of GAP-43 in ATF-3 positive neurons was observed immediately at day 3, but that became more pronounced from day 7 onwards. The distance of the axotomy site from the cell body seems to be important in determining GAP-43 expression and the speed of its up-regulation [18,48,49]. This could explain the delayed expression in OA animals, since the damage is far from the cell body. It should also be noted that GAP-43 was expressed in all cell size DRG cells, as occurs after peripheral axotomy [18]. The augmented expression of GAP-43 reinforces the hypothesis of nerve damage. ATF-3 along with other factors might be involved in the fate of these neurons after injury [13], and in some cases it might be having pro-survival, axonal-regeneration effect.

## Conclusions

In summary, MIA injection evokes dose-dependent expression of neuronal injury markers in DRG neurons, as well as a regeneration protein expression, indicating that axonal injury and a regeneration response may be happening in this model of OA. Two different phases were observed. In the initial days, a chemically-induced neuropathy may be occurring, possibly with the contribution of an inflammatory component, while at latter stages the mechanical activation of exposed primary afferents could be involved, but additional experiments are required to provide further evidence of this hypotheses. Actually, a neuropathic component in human OA has also been suggested [50-52]. Finally, correlation between this OA model and human disease must be cautious, because Barve et al. [53] analyzed and compared the transcriptional profile generated in OA rats induced by MIA with that of human OA and observed little similarity between the two.

## Methods

### Animals

Adult male Wistar rats (Charles River, France) weighing 230 ± 20 g were used in this study. Animals were housed in solid bottom cages, with water and food *ad libitum*, and animal room was kept at a constant temperature of 22 °C and controlled lighting (12 h light/12 h dark cycle).

All experimental procedures were performed in accordance with the ethical guidelines for the study of experimental pain in conscious animals [54], as well as the European Communities Council Directive 86/609/EEC, with all adequate measures being taken to minimise pain or discomfort of the animals.

### Induction of osteoarthritis

Under brief isoflurane anaesthesia, animals were injected intra-articularly with 0.3, 1 or 2 mg of MIA (Sigma-Aldrich, USA) dissolved in 25 μl of saline, or with only 25 μl of saline (controls). The syringe was inserted through the patellar ligament into the intra-articular joint space of the left knee. Animals were randomly assigned to each group (n = 5/experimental group).

### Behavioural testing

Nociception was evaluated in all animals by the Knee-Bend and CatWalk tests as previously described [55]. Testing was performed blind at days 0, 3, 6, 10, 13, 17, 20, 24, 27 and 31 post-injection. However, the number of animals in the behavioural assessment was not the same at the different time-points, since animals were pulled out at each sacrifice time-point resulting in a progressively decreasing number of animals, up to a minimum of 5 animals per group at day 31. Briefly, the Knee-Bend test consists on the recording of the squeaks and/or struggle reactions in response to five flexions and five extensions of the knee joint. The score of the test is determined by the type of reaction to each movement of the joint, according to the following evaluation scale: 0 - no responses to any kind of extension or flexion of the joint; 0.5 - struggle to maximal flexion/extension; 1 - struggle to moderate flexion/extension and also vocalizations to maximal flexion/extension; 2 - squeak reactions to moderate manipulations of the joint. The sum of the animal reactions, giving maximal values of 20, represents the Knee-Bend score, an indication of the animal’s movement-induced nociception.

For the CatWalk test, animals were placed in a glass platform illuminated such as to reflect light downwards at the points of contact of the paw with the surface, resulting in a bright sharp image of the paw-print. The platform was monitored by a video camera placed under the platform and connected to a computer equipped with video acquisition software (Ulead Video Studio, Corel Corporation, Canada). The intensity of the signal depends on the area of the paw in contact with the platform and increases with the pressure applied by the paw. Image J 1.37 (http://www.tucows.com/preview/510562) was used to analyse the images obtained from the videos recorded during the animal evaluation. The number and intensity of pixels above a defined threshold were quantified, allowing the comparison of the area/pressure applied by each paw. Results were expressed in total intensity of the ipsilateral hind paw as a percentage of the total intensity of both hind paws. Therefore, as alterations in the ipsilateral limb are in fact made up by the contralateral hindlimb, the weight applied to the contralateral paw may be inferred.

### Tissue processing

Seven days prior to the end of the experiments 5 μl of 2% Fluorogold (FG) (Fluorochrome, Denver, CO, USA) was injected intra-articularly in the left knee of the animals. This difference in the timing of FG injection was done to ensure the same migration time of FG in all animals, but, as a consequence animals sacrificed at day 3 had the FG injection before the MIA injection, as opposed to the others. At 3, 7, 14, 21 and 31 days after saline or 0.3, 1 or 2 mg of MIA injection, animals were anaesthetised and perfused with 4% paraformaldehyde with 0.1% of picric acid. Their DRG from lumbar segments 3, 4 and 5 (L3, L4 and L5) were dissected, post-fixed for 4 hours in the same fixative and kept in 30% sucrose with 0.01% sodium azide. DRG were serially sliced in 12 μm sections using a cryostat (Microm International GmbH, Germany), and every 10th section was collected in the same glass slide (8–10 sections from each DRG, on average). DRGs were oriented to ensure that longitudinal sections were made, and the number of sections obtained from each DRG was similar between animals, giving an indirect measure of this consistency. The cutting process was always performed by the same person and with the same method to ensure the consistency of the procedure throughout the study. The injected knees were also dissected, post-fixed for 72 hours and then decalcified for 8 hours in a buffer containing 7% AlCl3, 5% formic acid and 8.5% HCl, as previously described [55]. The joints were then washed in 0.1 M phosphate buffer saline (PBS) pH 7.2, and kept in 30% sucrose with 0.01% sodium azide until they were cut in 20 μm sections using the cryostat.

### Histological analysis of the knee joint

Knee joint sections were stained by the Fast Green and Safranin-O method in order to evaluate the extent of the histopathological lesions. Slides were mounted with Eukitt (Kindler, Germany) and images acquired with an Axioskop 40 microscope equipped with an AxioCam MRc5 camera (Carl Zeiss MicroImaging).

### Immunohistochemistry

Slides containing every tenth section of L3, L4 and L5 DRG of animals injected with saline or 0.3, 1 and 2 mg of MIA and sacrificed at 3, 7, 14, 21 and 31 days post-injection were used for immunofluorescence reactions for ATF-3 and NPY. Slides from animals injected with 2 mg of MIA were also used for double immunofluorescence reactions for ATF-3 and GAP-43 at the same time points after MIA injection. The immunohistochemistry reactions for each marker were performed in adjacent sections. DRG sections were rinsed in 0.1 M PBS pH 7.4, followed by PBS + 0.3% triton-X (PBST), and incubated in 10% normal serum in PBST for 90 min. Sections were then incubated overnight at room temperature with one of the following antibodies: rabbit anti-ATF-3 (1:500, Santa Cruz Biotechnology Inc, USA); rabbit anti-NPY (1:2000, Sigma-Aldrich, EUA). For double immunofluorescence reactions, sections were incubated overnight at room temperature with the antibodies rabbit anti-ATF-3 (1:500, Santa Cruz Biotechnology Inc, USA) and mouse anti-GAP-43 (1:500, Chemicon, EUA). After thorough PBST washing, sections were incubated with Alexa-Fluor 568 donkey anti-rabbit secondary antibody (1:1000, Molecular Probes, USA) 1 h at room temperature. For double immunofluorescence reactions, sections were incubated with Alexa-Fluor 488 donkey anti-rabbit and Alexa-Fluor 568 donkey anti-mouse secondary antibodies (1:1000, Molecular Probes, USA) 1 h at room temperature. Slides were then rinsed in PBST followed by PBS, mounted with Prolong Gold Antifade medium (Molecular Probes, USA) and coverslipped. Negative control immunohistochemistry reactions, where the procedure was the same with the exception of the absence of the primary antibodies, were performed to test for the specificity of the primary antibodies. Slides were viewed using a Zeiss Imager.Z1 fluorescence microscope (Carl Zeiss MicroImaging GmbH, Germany) and all neurons were identified based on morphological criteria and, in the ATF-3 reaction, counted as positively labelled only if the immunostained nucleus was clearly darker than the surrounding cytoplasm. Countings were done blind as to the experimental group and always by the same experimenter to assure reproducibility.

Cell size distribution was determined in the animals injected with 2 mg of MIA, by measuring their cross sectional area.

### Statistics

Results are presented as mean ± SEM. For behavioural data, the temporal profile evaluation was analysed by Repeated Measures ANOVA, followed by the Fisher's least significant difference (LSD) post-hoc test. Immunohistochemical data obtained from OA animals was compared with data from control animals using the Mann–Whitney test. A *P* value < 0.05 was accepted as statistically significant.

To examine the correlation between the behavioural measurements and ATF3 expression or NPY expression, the Pearson’s correlation coefficient was calculated by comparing the number of ATF3 or NPY in L3, L4 and L5 DRGs versus the Knee-Bend score and the ipsilateral paw print intensity measured by the CatWalk, at all time-points and for the three MIA doses.

## Competing interests

The authors declare that they have no competing interests.

## Authors’ contributions

JFG conceived and designed the experiments, conducted tissue processing, analysed the data, interpreted the results and participated in the drafting of the manuscript. SA contributed to the experimental design, conducted the histology and the statistical analysis and helped draft the manuscript. RS performed the immunohistochemistry analysis and helped draft the manuscript. MM performed the behavioural testing and helped draft the manuscript. JCL conceived and designed the experiments, interpreted the results, supervised the drafting of the manuscript and oversaw the overall execution of the project. All authors read and approved the final manuscript.
